# Analysis of the Capacity of the Fenton Process for the Treatment of Polluted Wastewater from the Leather Dyeing Industry

**DOI:** 10.1155/2023/4724606

**Published:** 2023-04-06

**Authors:** Carlos A. Gómez, Miguel-Ángel Gómez-García, Izabela Dobrosz-Gómez

**Affiliations:** ^1^Laboratorio de Materiales y Procesos Reactivos (LM&PR), Grupo de Investigación en Procesos Reactivos Intensificados y Materiales Avanzados (PRISMA), Departamento de Ingeniería Química, Facultad de Ingeniería y Arquitectura, Universidad Nacional de Colombia, Sede Manizales, Campus La Nubia, Apartado Aéreo 127, Manizales, Caldas, Colombia; ^2^Laboratorio de Materiales y Procesos Reactivos (LM&PR), Grupo de Investigación en Procesos Reactivos Intensificados y Materiales Avanzados (PRISMA), Departamento de Física y Química, Facultad de Ciencias Exactas y Naturales, Universidad Nacional de Colombia, Sede Manizales, Campus La Nubia, Apartado Aéreo 127, Manizales, Caldas, Colombia

## Abstract

In this work, the capacity of the Fenton oxidation process for the degradation of color and organic matter contained in the wastewater generated in the leather dyeing stage (WWDS) of an industrial tannery was evaluated. The wastewater characteristics included, among others, high toxicity (lethal concentration for *Artemia salina*, 24 h test, 50% of population = 93.71 ppm), high dye concentration (36 mg/L, yellow color), high chromium concentration (3.34 mg/L), and low biodegradability index (BOD_5_/COD ratio = 0.083). From an experimental design, the response surface methodology, and the multiobjective optimization analysis, the following optimal operating conditions were established: initial pH = 3.15, [Fe^2+^] = 0.981 mM, and [H_2_O_2_] = 5.38 mM. After 10 min of oxidation (determined from kinetic studies), it reached approximately 97% decolorization, COD reduction of approximately 82%, and TOC mineralization of approximately 92%. A synergistic effect of Fenton's reagents for TOC removal (*S*_TOC_ = 0.8) and decolorization (*S*_CN_ = 0.28) of the WWDS under study was confirmed experimentally. An increase in the biodegradability index, to a value of approximately 0.3, was confirmed. The cost of the treatment was estimated at 0.0112 USD/m^3^. Thus, the Fenton oxidation process allowed compliance with current Colombian environmental regulations and considerably improved the biodegradability and toxicity characteristics of the studied industrial effluent. It can be considered as an efficient alternative, easy to carry out on an industrial batch scale, and economically viable for the treatment of wastewater from the leather dyeing stage of an industrial tannery.

## 1. Introduction

Leather and its derivatives are among the most traded products in the world, generating a cash flow of more than 80 billion dollars a year [1]. Although 54% of the world's leather production is concentrated in China, Italy, Korea, India, Russia, and Brazil, a large part of its production chain has gradually migrated to developing countries [2]. For example, in Colombia, cattle raising (eighth country in the world) has generated adequate conditions for the growth of the leather industry. In fact, for the second half of 2021, the “sector of leather, leather tanning and other types of related industries” reported a growth of approximately 118% compared to the same period in 2020 [3].

The leather manufacturing is a chemical process that is carried out on skin of animal origin that fulfills the function of natural biological material. This involves the use of a large number of chemical agents and water to make it a product resistant to rot, heat, fire, and water and with characteristics of color, texture, elasticity, and durability. The integral process carried out in a tannery encompasses at least 25 different unit operations. Each of them generates wastewater with specific characteristics. It has been estimated that, on a global scale, the amount of wastewater produced by the tanning industry exceeds 600 million cubic meters per year [4]. For every ton of leather processed, around 50 m^3^ of wastewater is generated [5] which, depending on the operation unit, presents a high level of salinity, organic load, suspended solids, ammonium, nitrogen, chlorides, sulphides, chromium, dyes, and heavy metals, among others [6].

Wastewater generated by the tanning industry can be classified into five groups based on its production stage: (i) degreasing, (ii) liming/waxing, (iii) tanning, (iv) oiling, and (v) dyeing [7]. Recent studies have shown the advantages of separately treating the wastewater generated at each stage of the process and/or operating unit [8]. Among them are (i) lower pollutant load to be treated, (ii) lower operating costs, and (iii) the possibility of reusing the treated effluent in the specific production stage. The effluent originating from the dyeing stage (WWDS) is considered the bottleneck during the treatment of wastewater from tanning, since it contains the largest number of recalcitrant substances. In fact, many researchers have made reports directed at WWDS environmental risk and toxicity assessments confirming their negative impact when discharged without appropriate treatment into the environment [9]. Furthermore, industrial wastewater treatment is an important issue in the current context, not only to protect the environment from pollution (reducing and/or eliminating pollutants) but also to conserve water resources, reduce water stress, and promote the sustainability. Only approximately 3% of the earth's water is fresh, mostly locked up in glaciers, polar ice caps, atmosphere, and soil, and approximately 0.5% is available as freshwater (v.g., lakes, rivers, etc.) that must be shared between natural biota and human demands [10]. Although water is a continually renewable resource, its natural supplies are limited by the quantities that move through the natural water cycle. Residual dye is the main contaminant in WWDS. Dyes often contain chemical elements/compounds in their structures (e.g., benzidine, naphthalene, chromium, and other aromatic compounds) that make them toxic, carcinogenic, and mutagenic to various aquatic and terrestrial organisms and even humans (v.g., for azo dyes, the medium lethal dose, LD_50_, ranges from 100 to 2000 mg/kg body weight) [11]. Additionally, the disposal of untreated WWDS in a water source can stop its reoxygenation capacity causing eutrophication with alterations in the biology of aquatic ecosystems and interruptions in the photosynthetic processes of plants and algae (affecting the trophic chain even at concentrations of dye as low as 1 ppm) [12]. For these reasons, the development of viable and efficient treatment methods to reduce and/or eliminate the WWDS pollutant load has become a priority for the tanning industry.

The physicochemical characteristics of WWDS make its treatment difficult and highly specific. In fact, the primary treatment methods (for example, coagulation-flocculation, desorption, and filtration) present well-identified limitations such as phase change of contaminants and low removal of recalcitrant compounds and salts used during the tanning process; although, in some cases, they achieve good color and turbidity removal. In addition, biological (or secondary) processes are inefficient due to the low biodegradability of WWDS (in general, the (biochemical oxygen demand)/(chemical oxygen demand) ratio BOD_5_/COD is <0.4) and the presence of metals [13]. On the other hand, advanced oxidation processes (AOPs) are becoming emerging alternatives for the treatment of this type of wastewater [14] and effluents polluted with insecticides [15], chemicals used in plastics manufacturing [16], antibiotics [17, 18], azo dyes [19], and sewage sludge [20]. These processes are based on the production of the hydroxyl radical (HO^•^) (i.e., a short-lived, nonselective, and highly reactive oxidizing agent) that allows the mineralization of almost any organic and organometallic contaminant [21]. Among the most competitive AOPs at the industrial level is the Fenton oxidation process (FOP), whose performance in the treatment of complex wastewaters has proven to be efficient as well as easy to implement and does not require specialized equipment. There are several options for applying FOP, namely, homogeneous or conventional Fenton (i.e., liquid Fenton's reagents are added externally) [22], heterogeneous (v.g., based on solids containing iron and/or incorporating Fe onto surfaces of different carriers) [23], electro-Fenton (i.e., Fenton's reagents are generated *in situ*, electrically at the cathode and/or sacrificial anode) [24], and photoelectro-Fenton (i.e., the combination of HO^•^ generated *in situ* and the photolytic action of UV or solar irradiation) [25].

In general, FOP involves the presence of ferrous ion (Fe^2+^) and hydrogen peroxide (H_2_O_2_) in an acidic medium, for the formation of hydroxyl radicals (HO^•^, HO_2_^•^), through the following equations [26, 27]:

(1) $$\begin{array}{c} {Fe^{2+}+H}_{2}O_{2}⟶Fe^{3+}+OH^{-}+HO^{•}k=40-80\;M^{-1}s^{-1} \end{array}$$

(2) $$\begin{array}{c} Fe^{3+}+H_{2}O_{2}⟶Fe^{2+}+H^{+}+HO_{2}^{•}k=0.001-0.01\;M^{-1}s^{-1} \end{array}$$

Furthermore, Fe^2+^ can be regenerated by reaction (2).

The hydroperoxyl radical (HO_2_^•^) is also an oxidizing agent, but with a lower oxidation power than that of hydroxyl radical (HO^•^) [13]. Since the reaction represented in equation (2) is significantly slower than that described by equation (1), catalyst regeneration is considered the most critical stage of FOP. This reduces the rate of radical production and can eventually deplete the iron available for reaction (equations (3) and (4)) [28, 29].

(3) $$\begin{array}{c} Fe^{3+}+HO_{2}^{•}⟶Fe^{2+}+O_{2}+H^{+}k=1.2\times 10^{6}\;M^{-1}s^{-1} \end{array}$$

(4) $$\begin{array}{c} Fe^{3+}⟷F{eOH}^{2+}⟷Fe{\left(OH\right)}_{2}^{+}⟷Fe{\left(OH\right)}_{2}^{4+}{⟷Fe}_{2}O_{3}^{•}nH_{2}O \end{array}$$

Among the main advantages of the conventional Fenton for wastewater treatment are [25, 30] (i) simple and flexible operation that allows easy operation in existing plants, (ii) the use of easy-to-handle chemicals, (iii) low need for power input, (iv) it does not produce chlorinated compounds, and (v) a high capacity to remove color, to reduce the organic load (in terms of COD and/or total organic carbon (TOC) concentrations), as well as effluent toxicity. However, it is worth noting some drawbacks that hinder its large-scale applications [31]: (i) it can imply relatively high operating costs and safety problems related to the storage and transport of H_2_O_2_, (ii) large volumes of chemicals consumed to acidify wastewater prior to treatment and/or to neutralize treated solutions prior to final discharge and/or final disposal, (iii) accumulation of iron sludge that must be removed and/or properly disposed of at the end of the treatment, and (iv) general mineralization is not feasible due to the formation of Fe^3+^ complexes with generation of carboxylic acids that cannot be destroyed by bulk HO^•^. However, the conventional Fenton process has proven to be viable under normal temperature and pressure conditions and with rapid degradation kinetics that make it ideal for batch-scale effluent treatment.

In recent years, the conventional Fenton has been used in Colombia for the treatment of industrial wastewater of petrochemical [32] and textile origin [33], as well as from the production of soluble coffee [34]. However, to the best of our knowledge, it has not been used in the treatment of wastewater from the tannery industry. In most of the studies reported in the open literature for the treatment of tanning wastewater by FOP, the authors used synthetic (simulated) wastewater (i.e., dissolving dye in distilled water) [14, 35, 36]. Only a few reports can be found on the conventional Fenton application for industrial WWDS. Among them, Alashkar et al. [14] successfully implemented the homogeneous Fenton process to treat a highly contaminated WWDS (i.e., COD = 13120 mg O_2_/L, BDO_5_ = 6300 mg O_2_/L, and Cr = 260 mg/L) reaching COD and BDO_5_ removals of 99 and 98%, respectively. Unfortunately, in this work, no toxicity studies were performed nor were operating conditions optimized to reduce operating costs and maximize organic load removal. Therefore, in order to obtain information on the performance and applicability of conventional Fenton (hereafter just Fenton), further studies involving industrial tanning effluents are needed.

All the benefits of the Fenton process can only be realized by establishing its optimal operating conditions (e.g., pH and concentration of Fenton's reagents (Fe^2+^ and H_2_O_2_)) which depend on the type and concentration of contaminants in the effluent [12, 30]. Regarding the pH value, the chemistry of iron species requires acidic conditions (v.g., 2 < pH < 4) to prevent its precipitation as hydroxide and degradation of H_2_O_2_ into O_2_ and H_2_O. Concerning H_2_O_2_ concentration, it must be supplied on the basis of the chemical oxygen demand (COD) of the treated effluent. Finally, in relation to the ferrous ion, its high concentration leads to high reaction rates until its further increase seems to be marginal for the efficiency of the process. In addition, its excess can increase the total dissolved solids concentration, which will imply subsequent treatment steps and contribute to additional treatment costs.

The optimal operating conditions for Fenton oxidation can be determined through experimental design and the response surface methodology (RSM). RSM involves a group of mathematical and statistical techniques based on the adjustment of empirical models to the experimental data obtained according to the selected matrix [24, 37]. Linear or square polynomial functions are used to describe the performance of the studied system and to examine the experimental conditions until their optimization [38]. Its application as an optimization tool involves the following steps: (i) the choice of independent variables with important effects on the studied system (factors) and the definition of their experimental range (through preliminary experiments), (ii) the selection of dependent variables (responses), (iii) the selection of the experimental design and its experimental running, (iv) the statistical analysis and mathematical treatment (fitting to a polynomial function) of the experimental data obtained, (v) the evaluation of the fitted model, (vi) the optimization, and (vii) the experimental validation of the model.

In this work, the capacity of the Fenton oxidation process in the degradation of color and organic matter contained in an industrial effluent generated in the leather dyeing stage was evaluated. The optimal operating conditions of the Fenton process were defined through (i) a preliminary study that allowed defining the operating intervals of the most significant factors for the Fenton process (pH, [Fe^2+^] and [H_2_O_2_]), (ii) an experimental design (Box–Behnken type) and the statistical analysis of the results obtained, and (iii) the application of RSM and numerical multiobjective optimization tools that included the analysis of the operational costs of the process as a complementary criterion.

## 2. Methodology

In this experimental research, the raw WWDS corresponded to a representative sample from an industrial effluent from the dyeing stage of a tannery located in the department of Nariño (southern Colombia, South America). To determine its representative characteristics, the sampling was carried out quarterly. The first two samplings were performed in the period of time between September 2019 and January 2020 and the next two between September 2020 and January 2021 (that was a total of four samplings). The interruption in the sampling sequence (in March 2020) was due to the preventive isolation of people ordered by the Colombian government as a consequence of the COVID-19 pandemic. In each sampling, an integrated sample was taken, mixing three specific samples of 12 L each from different drums. They were taken manually, simultaneously, at the discharge point of each drum (that was a total of 36 L sampled each time). Thus, in total, during 4 samplings, 144 L of raw WWDS was taken. These were used as follows: (i) the determination of the physical-chemical parameters of the raw WWDS, developed after each sampling, in the shortest possible time, using approximately 15 L of the effluent (in total, approximately 60 L) (average characteristics of the raw WWDS are presented in Table 1); (ii) the development of preliminary tests with replicates (approximately 15 L); (iii) the run of the experimental design with replicates (approximately 10 L); (iv) the development of the kinetic study with replicates (approximately 10 L); and (v) the development of the Fenton process at optimal operating conditions for characterization purposes (approximately 20 L) (results are found in Table 1). The protocols for sampling, preservation, and management followed the guidelines of the Institute of Hydrology, Meteorology and Environmental Studies [40, 41] and the Standard Methods [42]. The analytical methods, experimental setup, and the experimental design and analysis of the results are detailed in the following sections.

### 2.1. Analytical Methods

The baseline of this study corresponded to the characterization of the physicochemical parameters of the industrial effluent according to current environmental regulations in Colombia [39]. Industrial (raw WWDS) and posttreatment effluents were analyzed in triplicate to establish the extent of treatment. Standard methods were followed for the quantitative analysis of different physicochemical parameters of the raw and treated effluent [42]: pH (4500 H^+^ B), chemical oxygen demand (COD) (5220 D), biochemical oxygen demand (BOD_5_) (5210 B), total suspended solids (TSS) (2540 D), total settleable solids (SSED) (2540 F), fats and oils (5520 D), methylene blue active substances (SAAM) (5540 C), total hydrocarbons (HTP) (5520 F), orthophosphates (P-PO_4_^3−^), and total phosphorus (4500 P-D), nitrates (N-NO_3_^−^) (4500 NO_3_^−^ A, B), ammoniacal nitrogen (N-NH_3_) (4500 NH_3_ C), total nitrogen (N) (4500 -N), chlorides (Cl^−^) (4500 Cl^−^ B), sulfates (SO_4_^−^) (4500 SO_4_^2−^ E), chromium (Cr) (3111 B), total acidity (2310 B), total alkalinity (2320 B), calcium hardness (3500-Ca B), total hardness (2340 C), true color (ISO 7887: 2012-04 B), total organic carbon (TOC) (5310 B), conductivity (2510 B), hexavalent chromium (3500-Cr B), dissolved oxygen (4500-O C), apparent color (2120 B), and real color (2120 B). The wavelength at which maximum absorption occurs was defined as a function of the absorption coefficient, measured with a NANOCOLOR® UV/Vis II spectrophotometer (Macherey-Nagel GmbH & Co. KG, Düren, Germany), by continuous scanning (every 2 nm) along the UV-Vis spectrum (200–800 nm wavelength). The dye concentration (mg/L) was determined by measuring the absorbance at the dominant wavelength of the dye (425 nm). All reagents were used as received from suppliers without any further purification. Their aqueous solutions were prepared using ultrapure water (Milli-Q system, Billerica, Massachusetts; conductivity = 1 *μ*S/cm). Sludge generation was quantified gravimetrically (XB 220A Precisa, Mettler-Toledo, Barcelona, España) following the standard 2540 D method [42].

The residual H_2_O_2_ concentration (for the experimental run carried out under optimum operating conditions) was measured by iodometric titration based on the oxidation of iodide to iodine in the presence of the molybdate catalyst. The iodine formed was then titrated with a thiosulfate solution using a starch indicator. This method was chosen considering its lower susceptibility to interference from organic compounds. Specifically, 15 mL of 20% sulfuric acid (H_2_SO_4_, 98%, 1.84 g/cm^3^, Merck KGaA, Darmstadt, Germany) solution (to ensure acidic conditions, pH≈4), 2 mL of 10% potassium iodide (KI, ≥99.99% trace metals basis, Merck KGaA, Darmstadt, Germany) solution, and 2∼3 drops of ammonium molybdate (NH_4_)_2_MoO_4_, 99.98% trace metals basis, Merck KGaA, Darmstadt, Germany) solution were added to 25 mL of WWDS sample and stored for 5 min in the dark. The sample was then titrated with 0.1 N sodium thiosulfate (Na_2_S_2_O_3_, ≥99.99% trace metals basis, Merck KGaA, Darmstadt, Germany) to a faint pale-yellow endpoint. Then, approximately 2 mL of starch indicator (starch soluble GR for analysis, Merck KGaA, Darmstadt, Germany) was added to the solution, and the titration was continued until a deep blue color turned colorless at the end point of the titration. The general stoichiometry of the reaction is that 1 mol of H_2_O_2_ reacts with 2 mol of Na_2_S_2_O_3_. Verification of the method was carried out by titrating a standard solution of hydrogen peroxide (H_2_O_2_, 30% m/v, BioPharm Chem, Darmstadt, Germany) at a concentration of 5.38 mM, and the measurement error was found to be ±3%.

Molecular weight distribution (MWD) analysis of the raw and Fenton-treated effluent was performed by membrane ultrafiltration. A stirred cell (Amicon, Model 8400, Merck KGaA, Darmstadt, Germany), containing cellulose ultrafiltration membranes (Millipore® Ultracell, Merck KGaA, Darmstadt, Germany) with nominal molecular weight limits (NMWLs) of 30, 10, 5 and 1 kDa, was used. The ultrafiltration operating pressure (0.4 MPa) was provided by a constant supply of highly pure nitrogen (N_2_, 99.999 vol. %, Praxair, Linde, Bogotá, Colombia). Before use, the membranes were washed for 30 minutes with 0.1 M sodium hydroxide (NaOH, ≥97% m/m, Carlo Erba, Val de Reuil, France) and rinsed with deionized water, according to manufacturer's instructions. The TOC of each molecular weight fraction was evaluated for permeates. Acute toxicity tests were performed using the crustacean *Artemia salina* (supplied by the Carolina Biological Supply Company, Burlington, NC, USA) according to the procedures of ISO 6341 : 2012 [43] and NTC-ISO 5667-16 [44]. The methodology proposed by Lieberman [45] and Maurer-Jones et al. [46] and the recommendations of Vanhaecke et al. [47] and the Carolina Biological Supply Company on procedures for sterilization and cleaning were followed. *Artemia salina* eggs (approximately 40 mg) were incubated (at 30°C) for 48 hours in an artificial aqueous medium containing 37 g/L of sea salt, illuminated by an 8 W lamp (Luminex®, Thermo Fisher Scientific, Waltham, MA, USA) that provided approximately 3500 lm of light intensity. This allowed the hatching of the eggs and the maturation of the nauplii. To determine the median lethal concentration (LC_50_) of an analyte (i.e., the concentration which causes the death to 50% of the population), 8–12 nauplii (N_O_) were transferred with a Pasteur pipette to each test tube (10 tubes, each 16 mm in diameter), previously washed and sterilized. Next, saline solution was added to each test tube, which contained 8–12 nauplii, to adjust 5 mL of total volume. Subsequently, the analyte in each tube was diluted to obtain a desired concentration. A blank test was also developed to determine if any external factor affects the nauplii. Each test was performed in triplicate for raw and treated wastewater. Finally, 24 h later, the number of living individuals (*N*_*t*_) in each test tube was quantified. The nauplius was considered dead if its appendages did not show movement for more than 10 s. The lethality in each test tube, corresponding to a different concentration of analyte, was expressed as a percentage of mortality:

(5) $$\begin{array}{c} Mortality\;\left(\%\right)=\frac{N_{O}-N_{t}}{N_{O}}\;x\;100\text{.} \end{array}$$

The tests were considered consistent if the mortality in the control (blank) did not exceed 10% under the validation of the probit model with a significance level of *p* < 0.05.

### 2.2. Experimental Setup

**Figure 1:**
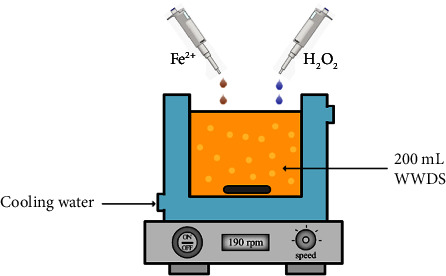
Diagram of the experimental setup to carry out the tests of the Fenton process.

Figure 1 presents a schematic of the experimental configuration used for the evaluation of the capacity of the Fenton process for the treatment of WWDS. To treat 200 mL of WWDS, a 250 mL borosilicate glass jacketed reactor was used. The temperature was kept constant at 20°C using an F-12 thermostatic bath (Julabo GmgH, Seelbach, Germany) connected to the jacket of the reactor. In the upper part of it, a PT-100 sensor (±0.01 K) was installed to measure the temperature of the solution. The WWDS sample was stirred, at a constant speed of 190 rpm, with a 4 cm long bar magnet to ensure homogenization and minimize resistance to mass transfer.

Initially, the experimental pH value of 3 was adjusted with 1 N sulfuric acid (H_2_SO_4_, 98 vol. %, Merck KGaA, Darmstadt, Germany). The addition of iron sulfate (FeSO_4_·7H_2_O, ≥98% m/m, Duksan, Gyeonggi, South Korea) and hydrogen peroxide (H_2_O_2_, 30% m/v, BioPharm Chem, Darmstadt, Germany) to the reactor containing the WWDS sample initiated the Fenton reaction. The total reaction time was set to 60 minutes. At the end of each test, the treated effluent was filtered and characterized. Furthermore, it was neutralized with 1 N sodium hydroxide solution (NaOH, ≥97% m/m, Carlo Erba, Val de Reuil, France) to pH = 6 (i.e., Fenton + neutralization effluent). The effluent was neutralized to comply with Colombian regulations regarding the minimum permissible pH for wastewater discharges to surface water bodies and public sewers [39]. All experiments were performed in triplicate. Considering that residual H_2_O_2_ can contribute to COD and BOD_5_ measurements, causing errors in their quantification and/or hindering bacterial action in subsequent biological treatments [48], it was eliminated at the end of the reaction. The residual H_2_O_2_ was quenched with manganese (IV) oxide (MnO_2_, ≥99.99% trace metals basis, Merck KGaA, Darmstadt, Germany) prior to analysis of COD, BOD_5_, and toxicity. In all cases, the average values of three independent measurements of COD, BOD_5_, and toxicity are reported.

### 2.3. Statistical Design of Experiments

A multifactorial, three central points, Box–Behnken experimental design (BBD) was applied to determine the effect of operation factors (e.g., Fe^2+^ concentration, H_2_O_2_ concentration, and pH) on the performance of the Fenton process. As response variables, the percentage of color removal, %R_CN_, and operating costs, COpT, were chosen.

BBD belongs to a class of second-order rotating or quasirotating (with orthogonality characteristics) experimental designs based on three-level incomplete factorial designs [49]. These experimental designs are considered less expensive than their corresponding 3*k* designs. The number of experiments (*n*) involved in a BBD is defined as *n* = 2*k* (*k* − 1) + *C*_0_ (where *k* is the number of factors and *C*_0_ is the number of central points). For comparison, the number of experiments for a central composite design (CCD) is *n* = 2*k* + 2*k* + *C*_0_. The efficiency of the experimental design was also considered, defined as the number of coefficients in the estimated model divided by the number of experiments [50]. Of the following three-factor experimental designs, namely, CCD, Doehlert design (DD), BBD, and three-level full factorial design, BBD and DD are slightly more efficient than CCD and much more efficient than three-level full factorial designs. Another characteristic of BBD is the lack of combinations in which all factors are simultaneously at their highest or lowest level, which prevents experiments under extreme conditions. In this way, BBD will contain regions of poor prediction quality (such as missing corners). However, this property can be useful if the researcher must avoid extreme combined factors. It also prevents possible data loss in these cases. Considering all these characteristics, BBD was selected and applied in this study.

The selected levels for each operating factor were established from preliminary tests. The values of the response variables were determined by the following equation:

(6) $$\begin{array}{c} {\%\text{R}}_{CN}=\frac{{CN}_{in}-{CN}_{out}}{{CN}_{in}}\;x\;100\text{,} \end{array}$$

where CN is the color number. This was determined by measuring the absorbance of the effluent in each of the three wavelengths (Abs_i_) stipulated in the Colombian environmental regulations [39], that is, at 436 nm, 525 nm, and 620 nm, and using the following equations.

(7) $$\begin{array}{c} {SAC}_{i}=\frac{{Abs}_{i}}{Cell\;length}\text{,} \end{array}$$

(8) $$\begin{array}{c} CN=\frac{{SAC}_{436}^{2}+{SAC}_{525}^{2}+{SAC}_{620}^{2}}{{SAC}_{436}+{SAC}_{525}+{SAC}_{620}}\text{.} \end{array}$$

The COpT values were determined in USD per cubic meter of treated wastewater, according to the following equation:

(9) $$\begin{array}{c} COpT\left(\frac{USD}{m^{3}}\right)=\left(\begin{array}{c} 0.01\left(\frac{USD}{kg}\right)\times \text{LG}\left(\frac{kg}{m^{3}}\right)+0.53\left(\frac{USD}{m^{3}}\right)\times PHC\left(\frac{mL}{mL}\right)+0.15\left(\frac{USD}{kg}\right)\times SHC\left(\frac{kg}{m^{3}}\right)\\ +0.67\left(\frac{USD}{m^{3}}\right)\times AS\left(\frac{mL}{mL}\right)+0.34\left(\frac{USD}{kg}\right)\times HS\left(\frac{kg}{m^{3}}\right) \end{array}\right)\text{,} \end{array}$$

where LG, PHC, SHC, AS, and HS are the amounts of sludge generated (kg/m^3^), Fenton's reagents (H_2_O_2_ (mL/mL) and FeSO_4_.7H_2_O (kg/m^3^)), H_2_SO_4_ (mL/mL) (used to adjust the pH of the reagent medium), and NaOH (kg/m^3^) (used for neutralization), respectively. Each of these terms was multiplied by the value corresponding to its price according to information from the Colombian market for the year 2021 [1]. It is important to note that the cost associated with the sludge generated includes its handling, transportation, and final disposal in a municipal sanitary landfill.

The experimental results obtained for the response variables were adjusted to a second-order polynomial according to the following equation:

(10) $$\begin{array}{c} y_{i}=\beta_{0}+\overset{k}{\underset{i=1}{\sum }}\beta_{i}x_{i}+\overset{k}{\underset{i=1}{\sum }}\beta_{ii}x_{ii}^{2}+\overset{k-1}{\underset{i=1}{\sum }}\overset{k}{\underset{j=i+1}{\sum }}\beta_{ij}x_{i}x_{j}\text{,} \end{array}$$

where *y*_*i*_ is the response variable, *β*_0_ is the coefficient of the intercept, *β*_*i*_ are the linear coefficients associated with each factor, *β*_*ii*_ are the quadratic coefficients of interaction of the factors with themselves, and *β*_*ij*_ are the coefficients of interaction between the factors. The goodness of fit for each response variable was defined from the coefficient of determination (*R*^2^) and the adjusted coefficient of determination (*R*^2^_adj_). Using analysis of variance (ANOVA) and Pareto diagrams, the statistical significance of the effect of the factors, with themselves and between them, on the response variables was determined. Using the fitted polynomial models, a constrained multiobjective optimization analysis was performed to determine the operating conditions that simultaneously maximized color removal and minimized COpT. This mathematical problem was solved using a genetic algorithm (*gamultiobj* function) available in the MATLAB® software.

## 3. Results and Discussion

### 3.1. Wastewater Characterization

The average physicochemical characteristics of the raw WWDS are presented in Table 1. It also includes, for comparative purposes, the maximum permissible limits for wastewater discharges to surface water bodies and public sewage, for the manufacture of leather goods, leather tanning, and dressing, in accordance with Colombian environmental regulations [39]. The conductivity of the studied effluent was high compared to that of rainwater or drinking water (i.e., 5–800 *µ*S/cm) due to the presence of different soluble salts used during the tanning process. The chromium concentration value did not comply with Colombian environmental legislation (i.e., 1.5 mg/L). It comes from the tanning operation that precedes the dyeing process (carried out in the same rotating drum). In fact, even if the tanned leather is carefully washed and the water used to dilute the dye is fresh, traces of chromium may remain both inside the drum and on the tanned leather.

Thus, the total chromium concentration must be also monitored after the Fenton treatment. The color concentration was also high (approximately 36 mg/L, yellow color). The presence of residual dye in the WWDS infers a load of organic compounds reflected in its TOC and COD values (although the latter is below the maximum permissible limit [39]). The value of the BOD_5_/COD ratio equal to 0.083 (<0.22) implied a low biodegradability index (BI) of the raw effluent [12].

Valuable additional information on the Fenton oxidation treatment can be obtained by analyzing the average oxidation state (AOS) of the raw and treated samples. The AOS can be calculated as follows [29, 51]:

(11) $$\begin{array}{c} AOS=4-1.5\times \frac{COD\left(mg/L\right)}{TOC\left(mg/L\right)}\text{.} \end{array}$$

**Figure 2:**
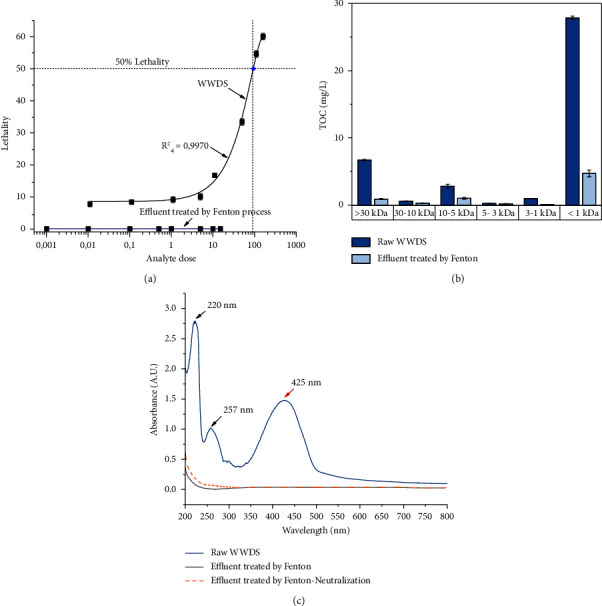
(a) Change in lethality for *Artemia salina* (24 h) of raw (WWDS) and Fenton-treated effluent, (b) MWD analysis of raw WWDS and Fenton-treated effluent in terms of TOC, and (c) UV-Vis absorption spectra before and after the Fenton and Fenton-neutralization process.

For the raw WWDS, the AOS of −2.25 indicated the presence of reduced compounds. In fact, the AOS can vary between −4 (the most reduced state of carbon, in the form of CH_4_) and +4 (the most oxidized state of carbon, in the form of CO_2_) [29, 44]. The WWDS characterization was complemented by an analysis of acute toxicity and that of MWD (Figure 2). The toxicity results on *Artemia salina* showed that, after 24 h, at least 60% of the individuals died when they remained submerged in raw wastewater. The lethal concentration for 50% of the population (LC_50_) corresponded to an effluent concentration of 93.71 ppm (Figure 2(a)). On the other hand, the stratification of the effluent according to the molecular size of the substances present, in terms of TOC concentration, showed that the main contributions of the pollutants are found among the molecular fractions >30 kDa (due to the presence of macromolecules such as fats and oils) and <1 kDa (e.g., perhaps due to the presence of acids) (Figure 2(b)).

The UV-Vis spectrum of raw WWDS consisted of three characteristic absorption bands. Two of them were in the UV region (Figure 2(c)): one at 220 nm and another at 257 nm. These may correspond to the set of chemical products used as auxiliaries in the preparation, dyeing, and finishing of leather. For example, sulfonates absorb in the region of 220–290 nm [52, 53]. Furthermore, carboxylic acids and aldehydes have also been found to absorb in the range of 190–280 nm [54]. The third characteristic absorption band was in the visible region, at 425 nm, and corresponded to the chromophore group of the dye.

### 3.2. Preliminary Tests

**Figure 3:**
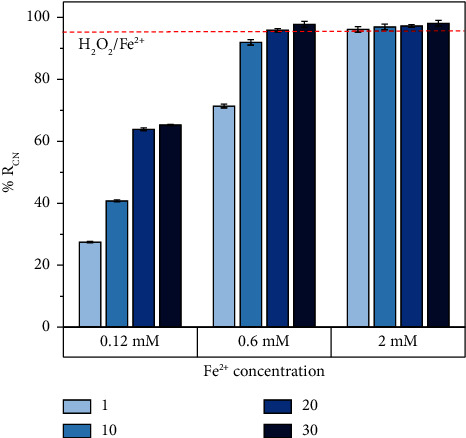
Effect of the H_2_O_2_/Fe^2+^ molar ratio on the percentage of color removal.

Initially, a series of preliminary experiments were developed to define the concentration ranges of Fenton's reagents to be evaluated (e.g., the operating variables that affect the performance of the Fenton process). The first decision criterion was related to the effect of the H_2_O_2_/Fe^2+^ molar ratio. According to Korpe and Rao [12], the value of this relationship defines different types of chemical processes in the reactive medium (e.g., H_2_O_2_/Fe^2+^ ≤ 0.5: chemical coagulation; H_2_O_2_/Fe^2+^ = 1.0: Fenton-like; H_2_O_2_/Fe^2+^ ≥ 1.0: catalytic oxidation). In this case, it was intended to prioritize the oxidative process over the coagulation process (since this latter does not allow the degradation of contaminants, but their precipitation and sedimentation). The H_2_O_2_/Fe^2+^ molar ratio ≥1 implies having an excess of H_2_O_2_ in the reaction medium. Based on the review of the literature for the treatment of wastewater contaminated with dyes within the yellow-red range [55–57], H_2_O_2_/Fe^2+^ ratios between 1 and 30 were initially chosen for evaluation. Additionally, the following operating conditions were established: (i) initial pH = 3.0, since it has been shown that the operational optimum of the Fenton process is close to this value; (ii) stirring speed = 190 rpm, temperature: 20°C, and reaction time = 60 minutes. The results obtained are shown in Figure 3.

H_2_O_2_/Fe^2+^ molar ratios in the range of 1–30 allowed greater than 95% color removals. On the other hand, at ferrous ion concentrations <2 mM, color removal increased with increasing H_2_O_2_ concentration, that is, when the H_2_O_2_/Fe^2+^ molar ratio increased. However, a molar concentration of Fe^2+^ equal to 0.12 mM was ineffective in terms of discoloration. It is worth noting that the final pH of the effluents treated using the Fenton process varied between 3.1 and 3.8, which implied the need for pH adjustment (e.g., neutralization with NaOH solution) after the treatment to guarantee compliance with current environmental regulations regarding this parameter (e.g., 6 < pH < 9 for wastewater discharges to surface water bodies and public sewage [39]). Furthermore, this pH adjustment will allow the remaining Fe^2+^ to precipitate in the reactive solution and will improve the final color of the treated effluent. Thus, neutralization tests were carried out up to pH = 6 and pH = 9. No significant differences in %RCN and COpT were observed as a function of final pH (i.e., after neutralization). Consequently, it was decided to neutralize the treated effluents to pH = 6. For the abovementioned reasons, the Fe^2+^ concentration range of 0.3 mM–2 mM was selected for the experimental design. Similarly, the H_2_O_2_ concentrations were chosen to satisfy the H_2_O_2_/Fe^2+^ molar ratio between 1 and 30. In addition, the initial pH range between 3 and 7 was chosen. Finally, the treatment of the WWDS by the Fenton process involved the following steps: (i) pH adjustment, (ii) Fenton oxidation reaction, and (iii) neutralization.

### 3.3. Experimental Design

Table 2 shows the matrix of the programmed experimental design (e.g., the experimental range evaluated for the three factors: pH, Fe^2+^ concentration, and H_2_O_2_ concentration) and the results obtained for the selected response variables (%R_CN_ and COpT) both after the Fenton process and after the Fenton neutralization (Fenton effluent at pH = 6). The %R_CN_ ranged between approximately 3.7% and 99% and the COpT varied between 0.0037 and 0.0332 USD/m^3^. At pH = 3 (theoretical optimum for the Fenton process), regardless of the value of the other two factors evaluated, the discolorations exceeded 96%. Some combinations of Fe^2+^ and H_2_O_2_ allowed similar results at pH = 5, but at significantly higher COpT (e.g., test 4). The results of the experiments carried out at pH = 7 generated the lowest %R_CN_.

### 3.4. Polynomial Models and Analysis of Variance

The experimental results of each of the response variables were correlated using second-order polynomial models obtaining the following equations:

(12) $$\begin{array}{c} \%R_{CN}=-21.3067+30.5969\;x\;pH+86.8564\;x\left[{Fe}^{2+}\right]+3.1804\;x\left[H_{2}O_{2}\right]-5.0225\;x\;{\left(pH\right)}^{2}-25.8097\;x\;{\left[{Fe}^{2+}\right]}^{2}-0.0993878\;x\;{\left[H_{2}O_{2}\right]}^{2}\text{,} \end{array}$$

(13) $$\begin{array}{c} COpT\;\left(\frac{USD}{m^{3}}\right)=0.000134942+0.0000125\;x\;pH+0.00824827\;x\left[{Fe}^{2+}\right]+0.000544388\;x\left[H_{2}O_{2}\right]-0.000003125\;x\;{\left(pH\right)}^{2}+0.0000519031\;x\;{\left[{Fe}^{2+}\right]}^{2}+6.37755\;x\;10^{-8}\;x\;{\left[H_{2}O_{2}\right]}^{2}\text{.} \end{array}$$

**Figure 4:**
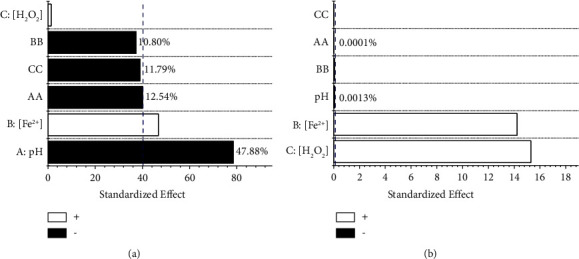
Pareto diagrams for (a) %R_CN_ and (b) COpT.

The analysis of variance (ANOVA) was performed using Fisher's test to determine the statistical significance of each of the factors and their interactions on the response variables (Tables 3 and 4). A high *F* value and a low *p* value (<0.05) imply that the variations in responses are statistically significant (with a confidence level of 95%). Additionally, the values of the correlation coefficients (*R*^2^ and *R*^2^_adj_ > 80%) demonstrated the good correlation of the models for the response variables [50]. The results obtained from the ANOVA (Tables 3 and 4) were consistent with others reported in the literature for the Fenton process [22, 31]. Several authors agree that pH is the most important factor in the production of hydroxyl ions. In fact, the pH value can influence more than any other reagent in reducing the color of the effluent [12]. Regarding COpT, ANOVA showed that, although there is a statistical significance for pH, [Fe^2+^], and [H_2_O_2_] (*p* value <0.05); the *F*-ratio for Fenton's reagents (Fe^2+^ and H_2_O_2_) is approximately 35,000 times higher than the other effects (simple and interactions). Thus, in practical terms, the influence of pH is negligible for this response variable. The ANOVA was completed with Pareto diagrams for each response variable (Figure 4). These present a series of bars whose extensions reflect the frequency or impact of each factor (e.g., the longer the bar is, the greater the influence and effect of the factor on the response variable), regardless of whether the effect is positive (increasing the effect will cause an increase in the response variable) or negative (increasing the effect will cause a decrease in the response variable). In addition, a vertical line (dotted) was included in each Pareto diagram. This establishes a separator from which the statistically significant effects are found for each response variable (analogous to the *p* value < 0.05 and corresponds to the *t* value in Student's *t*-distribution with a confidence level of 95%). Thus, the statistically important factors correspond to all those values that exceed the vertical line (Figure 4). The percentage effect of each factor on the response variable was calculated using equation (14), where *b*_*i*_ corresponds to the value of the standardized effect for factor *i* [38].

(14) $$\begin{array}{c} P_{i}=\left(\frac{b_{i}^{2}}{\sum b_{i}^{2}}\right)\times 100\text{.} \end{array}$$

In the ranges of the factors studied, the most significant variable for color removal was pH (approximately 48%), followed by Fe^2+^ concentration (approximately 17%) and the pH-pH interaction (approximately 12.5%); however, the concentration of H_2_O_2_ and the interactions [Fe^2+^]-[Fe^2+^] and [H_2_O_2_]-[H_2_O_2_] were not statistically significant. On the other hand, the concentration of Fenton's reagents (Fe^2+^ and H_2_O_2_) represented approximately 99% of the effects in COpT.

### 3.5. Response Surfaces and Optimization

**Figure 5:**
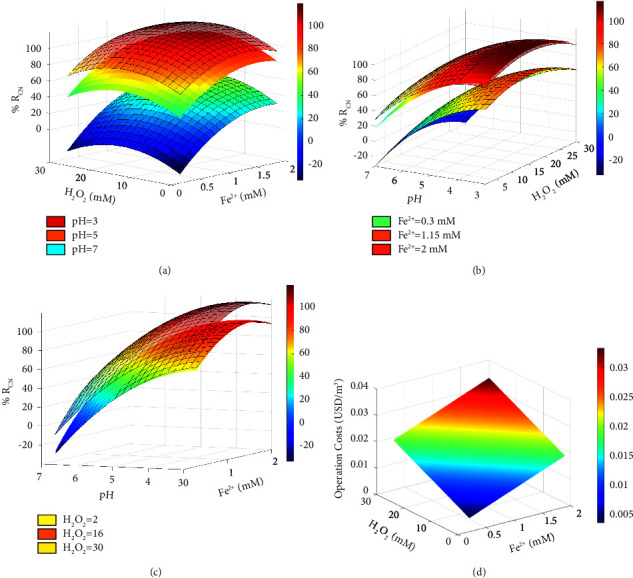
Three-dimensional plots of surface response for the interactive effect of pH, [Fe^2+^], and [H_2_O_2_] on % RCN and COpT.

With the fitted models (equations (12) and (13)), response surface diagrams were generated for the response variables (%R_CN_ and COpT) (Figure 5). As three factors were involved in each model (pH, [Fe^2+^], and [H_2_O_2_]), each surface was built fixing one factor and varying the other two.

In all surfaces generated as a function of the initial pH of the effluent (Figure 5(a)), there is a concave trend. Regardless of the pH value, an increase in the concentration of Fenton's reagents increased color removal up to a certain point (maximum), after which a further increase in reagent concentration became counterproductive for the efficiency of reaction (Figures 5(b) and 5(c)). In fact, when the pH has a value close to 3.0, the catalytic decomposition of H_2_O_2_ is faster, which suggests a greater production of HO^•^ radical and its reaction with organic contaminants (equations (15)–(17)) [12].

(15) $$\begin{array}{c} OH^{•}+RH\;⟶R^{•}+H_{2}O \end{array}$$

(16) $$\begin{array}{c} R^{•}+Fe^{3+}\;⟶R^{+}+Fe^{2+} \end{array}$$

(17) $$\begin{array}{c} R^{+}+OH^{-}⟶R-OH \end{array}$$

The increase in pH above 5.0 reduces the Fenton capacity due to the precipitation of Fe^3+^ as iron hydroxide and decomposition of H_2_O_2_. In addition, H^+^ deficiency hinders the formation of HO^•^ and decreases its oxidative power [19, 24]. Furthermore, high pH values can cause complexation reactions and precipitation of iron oxides, leading to the production of excess sludge. On the other hand, low pH values also negatively affect the Fenton oxidation process because (i) H_2_O_2_ can be unstable and can form H_3_O_2_^+^, which obstructs HO^•^ production, (ii) HO^•^ can be scavenged due to the excess of H^+^, (iii) it prevents the interaction between Fe^3+^ and H_2_O_2_, and (iv) [Fe(H_2_O)]^2+^ with low oxidative power can be formed [26, 31].

H_2_O_2_ constitutes the source of HO^•^ in the Fenton process, equation (1), and Fe^2+^ regeneration (equation (2)). However, at a high concentration of H_2_O_2_, it acts as a hydroxyl radical scavenger (equation (18)), producing hydroperoxyl radicals HO_2_^•^ [58], with a considerably lower oxidative capacity (Figure 5(c)) and giving rise to competitive reactions (equations (19) and (20)) [59].

(18) $$\begin{array}{c} OH^{•}+H_{2}O_{2}⟶H_{2}O+HO_{2}^{•} \end{array}$$

(19) $$\begin{array}{c} HO_{2}^{•}+OH^{•}⟶H_{2}O+O_{2} \end{array}$$

(20) $$\begin{array}{c} OH^{•}+OH^{•}⟶H_{2}O_{2} \end{array}$$

Regarding the effect of the Fe^2+^ concentration (Figure 5), it was observed that [Fe^2+^] = 0.3 mM is insufficient to generate a high color removal (>95%). However, as observed with H_2_O_2_, the excess of Fe^2+^ favors competitive reactions causing loss of the reagent and decreasing the concentration of the hydroxyl radical (equations (21)–(23)) [26].

(21) $$\begin{array}{c} OH^{•}+Fe^{2+}⟶Fe^{3+}+OH^{-} \end{array}$$

(22) $$\begin{array}{c} Fe^{2+}+HO_{2}^{•}\;⟶Fe^{3+}+OH^{-} \end{array}$$

(23) $$\begin{array}{c} Fe^{3+}+HO_{2}^{•}\;⟶Fe^{2+}+O_{2}+H^{+} \end{array}$$

It must be ensured that the Fe^2+^ ions supplied at the beginning of the reaction are completely consumed, since their residual presence will generate an increase in color and total suspended solids due to the precipitates generated by a chemical coagulation and/or neutralization process [58, 59].

Concerning COpT, as expected, the higher the amount and/or concentration of Fenton's reagents used, the higher the costs (Figure 5(d)). Therefore, the minimum COpT can be anticipated at the lower end of the experimental range. However, the least color reduction is obtained under such conditions. For these reasons, RSM did not allow defining operating conditions to simultaneously maximize %R_CN_ and minimize COpT. Thus, a multivariate optimization problem was formulated. It consisted of the determination of the minimum COpT achievable to reach the highest %R_CN_ value. It includes equations (12) and (13) and the following restriction:

(24) $$\begin{array}{c} Minimize\;COpT\left(\left.\left[H_{2}\left.O_{2}\right],\left[F\right.e^{2+}\right]\right)\right.\\ Maximize\;R_{CN}\;\left(\left[H_{2}O_{2}\right],\left[Fe^{2+}\right],pH\right)\\ subject\;to\text{: }97\leq R_{CN}\leq 100\text{.} \end{array}$$

The optimization problem was solved using a genetic algorithm (*ga* function available in the MATLAB® software). As the response surface corresponding to the COpT is flat near its minimum value and it is also a discrete variable (its minimum denomination is 0.01 USD), there is the possibility of detecting multiple optimal values. The results obtained together with their experimental verification (developed in triplicate) are presented in Table 5.

The residual concentration of H_2_O_2_ (85.9 ± 3.1 mg/L), determined by iodometric titration, implies that 53% of H_2_O_2_ fed in the process was actually used during the Fenton process. Of the two Fenton's reagents, H_2_O_2_ appears to be the most critical considering that it directly affects the theoretical amount of HO^•^ radicals formed. Moreover, the H_2_O_2_ dosage would be highly dependent on the initial COD, and a high initial COD would generally require more H_2_O_2_. The theoretical mass ratio of removable COD to H_2_O_2_ is 470.6/1000. In other words, 1000 mg/L of H_2_O_2_ can theoretically eliminate 470.6 mg/L of COD by oxidation. Therefore, the H_2_O_2_ efficiency (%*η*) can be defined as follows [60]:

(25) $$\begin{array}{c} \%\eta =\frac{{COD}_{oxid}}{0.4706\;x\left[H_{2}O_{2}\right]}\;x\;100\text{,} \end{array}$$

where COD_oxid_ is the COD removed by oxidation and [H_2_O_2_] is the dosage of hydrogen peroxide. For the WWDS studied, %*η* corresponded to 174% (considering 150 mg/L of COD removed and H_2_O_2_ dose of 183 mg/L (5.38 mM)). The %*η* value above 100% was previously reported for the untreated leachate, at a low H_2_O_2_ concentration relative to COD concentration [60, 61]. This suggests that organic compounds are mainly oxidized by HO^•^ radicals (formed from H_2_O_2_) rather than by H_2_O_2_ itself [62]. HO^•^ radicals can react with organic matter and/or Fenton catalyst through three routes: hydroxyl addition (equation (26)), hydrogen abstraction (equation (15)), and/or electron transfer (equation (21)) [63]. Hydroxyl addition can occur with organic compounds containing aromatic systems or carbon-carbon multiple bonds; hydrogen abstraction takes place with unsaturated organic chemicals, while electron transfer occurs if HO^•^ interacts with inorganic ions.

(26) $$\begin{array}{c} OH^{•}+R\;⟶\;{\left(ROH\right)}^{•} \end{array}$$

(27) $$\begin{array}{c} R^{•}+O_{2}\;⟶\;{ROO}^{•} \end{array}$$

HO^•^ radicals reacting with organic compounds can generate organic radicals (equation (15)) that react immediately with dissolved oxygen to yield peroxyl radicals (equation (27)), initiating subsequent oxidation chain reactions and leading to further decomposition and even eventual mineralization into water and CO_2._ Furthermore, the %*η* value above 100% could indicate that COD was removed by oxidation and coagulation, as previously suggested by Kang and Hwang [64]. On the other hand, the optimal concentration of ferrous ions is also important. This allowed maximizing the net production of HO^•^ radicals, avoiding their scavenging effect (v.g., equation (21)). An optimal H_2_O_2_/Fe^2+^ molar ratio for the Fenton oxidation of WWDS under study is 5.38. This suggests that catalytic oxidation (H_2_O_2_/Fe^2+^ ≥ 1.0) is prioritized during treatment [12]. In this study, the Fenton oxidation under optimal operating conditions allows reducing the COD concentration of WWDS by more than 92%, presenting COD values comparable to those of water in the absence of contaminants (<L.C. = 13.56 mg/L O_2_).

### 3.6. Kinetic Study

**Figure 6:**
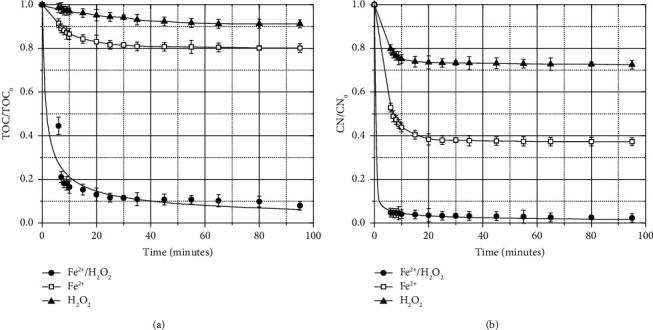
The evolution of (a) color (CN) removal and (b) TOC removal as a function of reaction time. Fenton operating conditions: initial pH = 3.15, [H_2_O_2_] = 5.38 mM, and [Fe^2+^] = 0.981 mM. The efficiency of Fe^2+^ alone ([Fe^2+^] = 0.981 mM) and H_2_O_2_ alone ([H_2_O_2_] = 5.38 mM) at pH = 3.15 is also included.

To determine the oxidation time required for the treatment of the WWDS studied, additional Fenton tests were developed under optimal operating conditions (Table 5). The variation of color and TOC concentrations as a function of time was controlled. The evolution of color (CN) and TOC, during 90 minutes of oxidation, is reported in Figure 6. The COD concentration decreased very quickly (during the first 6 minutes of reaction) below its quantification limit (<L.Q. (13.56)).

The color removal occurred quickly (e.g., the first six minutes of reaction account for more than 90% of the total degradation achieved during the test (Figure 6(a)). It is during this time interval that a greater amount of HO^•^ is produced (equation (1)), which reacts with the organic contaminants in the effluent and effectively degrades the color (equations (15)–(17)). However, the TOC reduction so far (e.g., the first six minutes of reaction) has only reached approximately 50% of the total degradation achieved during the test (Figure 6(b)). Continuous degradation is observed between 6 and 10 minutes of reaction, but at a slower rate. In fact, the production of ferric ions in the first minutes of reaction gives rise to a competitive reaction for H_2_O_2_, which decreases the amount of HO^•^ and produces the hydroperoxyl radical (with lower oxidating power (equation (2)) [57]. Although the reaction promoted by Fenton's reagents continues for a few more minutes, after 10 minutes of reaction, more than 97% of the color and TOC removals have already been achieved. Therefore, 10 minutes was considered a sufficient reaction time for the treatment of the WWDS by the Fenton process under optimal operating conditions. The experimental results, shown in Figure 6, were fitted to different reaction rate (degradation) expressions (power-law models for homogeneous reactions [16, 65, 66]) to fit both color-time and TOC concentration-time data. A nonlinear regression method of data analysis (using *nlinfit*, *nlparci*, and *nlpredci* functions available in MATLAB® software) allowed the determination of the values of the kinetic parameters (e.g., the reaction order and specific reaction rate constant), their confidence bands (dotted lines in Figure 6), and the uncertainties, as shown in the following equations.

(28) $$\begin{array}{c} r_{CN}=-\left(1.52\times 10^{-4}\pm \;6.41\times 10^{-5}\right)C_{CN}^{5.13\;\pm \;0.31}R_{adj}^{2}=0.999\text{,} \end{array}$$

(29) $$\begin{array}{c} r_{TOC}=-\left(1.17\times 10^{-3}\pm \;3.1\times 10^{-4}\right)C_{TOC}^{2.80\;\pm \;1.06}R_{adj}^{2}=0.936\text{.} \end{array}$$

The goodness of fit of the reaction rate laws to the experimental data is evidenced by the values of the determination coefficients. Likewise, the confidence bands demonstrate the reproducibility of the data and the confidence in the modeled behavior (Figure 6).

Figure 6 also presents the evolution of color (CN) removal (Figure 6(a)) and TOC removal (Figure 6(b)), as a function of reaction time, during WWDS treatment, using Fe^2+^ alone ([Fe^2+^] = 0.981 mM) and H_2_O_2_ alone ([H_2_O_2_] = 5.38 mM) at pH = 3.15. These experiments were performed to evaluate the decontamination ability provided by each Fenton's reagent, used separately, under similar operating conditions. During the first ten minutes of reaction, H_2_O_2_-assisted oxidation accomplished approximately 23% of color removal and only approximately 4% of TOC removal. On the other hand, in the presence of Fe^2+^, the color removal efficiency was approximately 57% and that of TOC was approximately 14%. These last removals are due to the reaction between FeSO_4_ and alkalinity (reported in Table 1) of the treated effluent (e.g., chemical coagulation). The combined Fe^2+^/H_2_O_2_ system, under optimized operating conditions, reached 97.5% and 92% of color and TOC removal, respectively, within 10 minutes of reaction. Regarding TOC removal, the rate constants of 0.05 × 10^−3^ ± 0.02 × 10^−3^ min^−1^, 0.18 × 10^−3^ ± 0.06 × 10^−3^ min^−1^, and 1.17 × 10^−3^ ± 3.1 × 10^−4^ min^−1^ were calculated for H_2_O_2_, Fe^2+^, and Fe^2+^/H_2_O_2_ systems, respectively. Regarding decolorization, the rate constants of 0.60 × 10^−4^ ± 0.04 × 10^−5^ min^−1^, 0.71 × 10^−4^ ± 2.98 × 10^−5^ min^−1^, and 1.52 × 10^−4^ ± 6.41 × 10^−5^ min^−1^ were determined for H_2_O_2_, Fe^2+^, and Fe^2+^/H_2_O_2_ systems, respectively. This indicates that the combined Fe^2+^/H_2_O_2_ system showed a better performance than the individual ones due to the production of hydroxyl radicals by Fenton reaction in solution, which implies a significant synergistic effect of Fe^2+^ and H_2_O_2_. As suggested by Hayati et al. [65], equation (30) can be used for the evaluation of the synergy index as follows:

(30) $$\begin{array}{c} S_{j}=\frac{k_{{Fe}^{2+}/\;H_{2}O_{2}}-\left(k_{{Fe}^{2+}}+k_{H_{2}O_{2}}\right)}{k_{{Fe}^{2+}/\;H_{2}O_{2}}}\text{,} \end{array}$$

where S_*j*_ corresponds to the synergy index of *j* parameter, and k_Fe^2+^_, k_H_2_O_2__, and k_Fe^2+^/ H_2_O_2__ correspond to the rate constants of the individual and integrated processes, respectively. Therefore, negative *S*_*j*_ values suggest an inhibition effect, zero value indicates a cumulative effect, and finally, a positive *S*_*j*_ implies a synergistic process. The synergy indexes calculated for TOC removal (*S*_TOC_ = 0.8) and decolorization (*S*_CN_ = 0.28) of WWDS using the Fe^2+^/H_2_O_2_ system confirmed the synergistic effect of Fe^2+^ and H_2_O_2_.

### 3.7. Characterization of the Treated Effluent

The effluent obtained after the Fenton oxidation treatment (carried out at optimal operating conditions for 10 minutes of reaction) was characterized, before and after its neutralization (pH = 6), in terms of the physicochemical parameters contemplated by the environmental Colombian legislation [39]. The results are presented in Table 1. Compared with the characteristics of the raw sample, the COD decreased by more than 92%, reaching values comparable to those of water in the absence of contaminants (<L.Q. = 13.56 mg/L O_2_). Although the exact COD value could not be measured (it was below its quantification limit), the COD decrease showed that the Fenton process was effective in terms of contaminant oxidation. BOD_5_ was reduced by more than 77% and TOC by more than 82%. Thus, the efficiency of the Fenton process to oxidize organic matter was demonstrated. The biodegradability index (BI = BOD_5_/COD) of the treated water could not be calculated accurately (COD values <L.Q. = 13.56 mg/L O_2_), but it increased more than 265% with respect to the raw effluent (0.083), reaching a value greater than or equal to 0.3 (the value by which the wastewater is considered biodegradable is BI > 0.22 [12]).

In addition, the AOS of the Fenton effluent increased from −2.25 (raw sample) to values greater than 1.13. This indicates a significant degree of oxidation of the organic substances originally present in the wastewater. The decrease in the concentrations of fats and oils (63%), nitrates (96%), ammoniacal nitrogen (8.8%), total nitrogen (29%), and chlorides (22%), total acidity (34%), total alkalinity (97%), total hardness (13%), and calcium hardness (33%) was confirmed (Table 1).

However, TSS, SS, sulfates, and conductivity increased compared to their respective values for raw wastewater (Table 1). In the case of the first two parameters, this can be attributed to the sludge generated during the Fenton process and the presence of the sulfate ion from Fenton's reagent (iron sulfate hepta-hydrate). However, the concentration of sulfates in the treated effluent is below 500 mg/L, this being the maximum value recommended by the World Health Organization for drinking water [67]. It should be noted that the treatment and disposal of the solids generated during the Fenton treatment process was considered as part of the COpT (equation (9)). The decrease in total chromium concentration deserves special attention, from 3.34 mg/L in WWDS (significantly above the Colombian environmental standard for wastewater discharges to surface water bodies and public sewage: 1.5 mg/L [39]) to a value below the detection limit of the method (<0.75 mg/L). This can be attributed to the neutralization step. In fact, alkaline precipitation has been considered as an effective strategy for the decontamination of wastewater containing chromium. Cr^3+^ and Fe^3+^ have similar ionic radii (i.e., 0.63 and 0.64 Å, respectively) [68]. Consequently, the released Cr^3+^ ions could be structurally incorporated into Fe(III) (oxy)hydroxides with the formation of the mixed Fe(III)-Cr(III) (oxy)hydroxides by adjusting the pH of the solution to 6.0 [69].

The results of MWD analysis for WWDS before and after Fenton treatment, in terms of the TOC concentration of each fraction, are compared in Figure 2(b). For the raw sample, approximately 88% of the TOC concentration corresponded to fractions >30 kDa (approximately 17%) and <1 kDa (approximately 71%). After the Fenton treatment, the organic load decreased by approximately 87% and 83% for these two fractions, respectively. In general, despite the fact that the contaminants decreased in all the fractions almost in the same proportion to the overall TOC reduction, the fraction with MW < 1 kDa presented the highest number of residual contaminants associated with the final TOC (67%). This is due to the fact that the oxidation of pollutants does not occur selectively and many of the larger substances present in wastewater broke down into smaller molecules (e.g., <1 kDa).

The UV-Vis absorption spectra of the effluents treated with Fenton and Fenton-neutralization are presented in Figure 2(c). Note that the three characteristic absorption bands determined for the WWDS practically disappeared. These results imply that the Fenton process allows the oxidation of organic contaminants present in the wastewater, including the dye. The fraction of the absorption band persistent in the UV region may be associated with the presence of residual carboxylic acids (also absorb in the 190–280 nm range [54]) and/or incomplete oxidation products. This coincides with the remaining values of TOC. According to Oturan et al. [70], tartaric, acetic, and fumaric acids are produced from oxidative cleavage of the benzene ring and aromatic byproducts. These can be further oxidized to oxalic and formic acids. However, since the chemical formula of the dye present in the WWDS under study is unknown, it is not possible to reach a definitive conclusion.

The toxicity of the Fenton effluent on *Artemia salina* (contact time 24 h) was evaluated for different concentrations (Figure 2(a)). The lethality decreased from LC_50_ = 93.71 ppm, for the raw sample, to a value of 0.00 ppm for the Fenton-treated effluent (e.g., no dilutions of the treated wastewater were found that cause the death of individuals in the population). This represents a 100% decrease in lethality. The toxicity study demonstrated the high capacity of the Fenton treatment method used in terms of reducing the environmental impact with the bioindicator used.

Table 6 presents a brief comparison of the results obtained in this study with those previously reported in the literature for the treatment of industrial tanning wastewater using FOP, based on its operating conditions and removal efficiencies. However, this comparison is not a simple task due to the substantial differences in the characteristics of the treated effluents as well as the Fenton operating conditions used. Regarding the efficiency of the treatment used for the degradation of dyes in a similar range of concentrations (e.g., concentration of dye and/or COD), similar results were obtained by both Bravo-Yumi et al. [71], who applied electro-Fenton and photoelectro-Fenton for the treatment of synthetic effluents polluted with the mixture of dyes (Violet RL-Green A), and by Lofrano et al. [36], who used photo-Fenton for the treatment of effluent containing syntan. Likewise, the novel Fenton oxidation, which applied the combination of Fe^2+^, H_2_O_2_, CaO, and graphene nano-oxide, was very effective for the treatment of an industrial tannery effluent [14]. However, a lack of information on the operating costs of those FOP treatments applied for both industrial and synthetic (simulated) tanning effluents makes fair comparison difficult. The evaluation in terms of the reaction rate constant is also not straightforward because the experimental data were fitted to different power-law models (e.g., pseudo-first order and n-order). On the other hand, it can be observed that the heterogeneous Fenton process, developed by applying activated carbon doped with Co oxide (1%) [35], was efficient in the removal of COD and TOC from industrial wastewater. However, the biodegradability index (BOD_5_/COD) of the treated effluent (0.08) was significantly lower than that of the raw effluent (0.38). Still, it is possible to conclude that the operating conditions of the Fenton treatment optimized in this study, using the experimental design, the response surface methodology, and the multiobjective optimization, allowed an economic and remarkable performance. In this way, the amount of sludge generated and the consumption of chemicals were reduced, aspects that are considered to be the main limitations of large-scale conventional Fenton oxidation. This is reflected in the competitive operating cost (0.0112 USD/m^3^) of this treatment. However, the requirement for an acidic operating pH and potential safety issues related to the storage and transport of H_2_O_2_ remains a challenge. The implementation of the Fenton process let to develop a simple, economical treatment system that is easy to implement and manipulate, taking advantage of the infrastructure usually available in Latin American tanneries.

## 4. Conclusions

The capacity of the conventional Fenton oxidative process for the degradation of color and organic matter contained in the wastewater generated in the leather dyeing stage of an industrial tannery was evaluated. The industrial effluent was characterized with a yellow color (approximately 36 mg/L), high chromium concentration (approximately 3.34 mg/L), the presence of reduced compounds (AOS = −2.25, with predominant fractions of compounds with molecular weights >30 kDa and <1 kDa), a low biodegradability index (0.083), and toxicity (with a lethal concentration LC_50_ of approximately 94 ppm using *Artemia salina* as a bioindicator). From the experimental design, the response surface methodology, and the multiobjective optimization analysis, the following optimal operating conditions were established: initial pH = 3.15, [Fe^2+^] = 0.981 mM, and [H_2_O_2_] = 5.38 mM. These conditions allowed maximizing the discoloration of the effluent (>97%) and minimizing the operational costs (0.0112 USD/m^3^). The kinetic analysis, carried out under optimal Fenton operating conditions, allowed to determine that 10 minutes of Fenton oxidation allowed reaching approximately 97% of color removal. The predictive capacity of the kinetic expressions was verified against the experimental data. A synergistic effect of Fenton's reagents for TOC removal (*S*_TOC_ = 0.8) and decolorization (*S*_CN_ = 0.28) of WWDS under study was confirmed experimentally. The treated effluent presented a chromium concentration below the maximum allowed by Colombian regulations. Furthermore, the BOD_5_ concentration was reduced by approximately 77%, the TOC in approximately 82%, the COD in more than 92%, and the biodegradability index increased from 0.083 to values higher than 0.3. The treatment complied with current national environmental regulations and considerably improved the biodegradability and toxicity characteristics of the effluent. Considering the results obtained and despite the drawbacks that the conventional Fenton process may present in large-scale applications, it is possible to conclude that, for the case analyzed, the Fenton oxidation can be considered as an efficient alternative, easy to implement and manipulate on a large-scale (batch mode), taking advantage of the infrastructure usually available in Latin America, and economically viable for the treatment of wastewater from the leather dyeing stage of an industrial tannery.

## Figures and Tables

**Table 1:** Average characteristics of the raw WWDS (mean value of 4 samples), generated in the dyeing stage of a tannery located in the department of Nariño, together with the permissible discharge limits established by Colombian legislation [39]. The average characteristics of the effluents treated by Fenton and Fenton + neutralization are also included.

Parameter	Units	Permissible limits^a^	Average value and deviations		
			WWDS raw effluent	Fenton effluent	Fenton + neutralization effluent
General					
pH		6.00 a 9.00	7.00 ± 0.8	2.97 ± 0.2	6.08 ± 0.05
Chemical oxygen demand (COD)	mg/L O_2_	1200.00	163.1 ± 40	<L.Q. (13.56)	<L.Q. (13.56)
Biochemical oxygen demand (BDO_5_)	mg/L O_2_	600.00	13.47 ± 2.3	3.04 ± 0.6	3.10 ± 0.5
Total suspended solids (TSS)	mg/L	600.00	58 ± 8	62.50 ± 7	65.10 ± 4
Settleable solids (SS)	mL/L	2.00	1.2 ± 0.3	1.3 ± 0.1	1.4 ± 0.1
Oil and grease	mg/L	60.00	2.34 ± 0.15	0.85 ± 0.03	0.84 ± 0.01
Methylene blue active substances (MBAS)	mg/L	A&R	<L.Q. (0.01)	<L.Q. (0.01)	<L.Q. (0.01)
Hydrocarbons					
Hydrocarbons (HTP)	mg/L	10.00	0.32 ± 0.01	0.32 ± 0.02	0.32 ± 0.01
Phosphorus compounds					
Orthophosphate (P-PO_4_^3−^)	mg/L	A&R	<L.Q. (0.09)	<L.Q. (0.09)	<L.Q. (0.09)
Phosphorus (P)	mg/L	A&R	<L.Q. (0.01)	<L.Q. (0.01)	<L.Q. (0.01)
Nitrogen compounds					
Nitrogen-nitrate (N-NO_3_^−^)	mg/L	A&R	11.41 ± 0.9	0.45 ± 0.02	0.47 ± 0.01
Nitrogen-ammonia (N-NH_3_)	mg/L	A&R	3.31 ± 0.25	3.02 ± 0.23	3.05 ± 0.18
Nitrogen (N)	mg/L	A&R	8.24 ± 0.72	5.83 ± 0.63	5.87 ± 0.54
Ions					
Chloride (Cl^−^)	mg/L	3000.00	420 ± 36	327.75 ± 27	314.49 ± 15
Sulfate (SO_4_^−^)	mg/L	A&R	267.70 ± 19	418.00 ± 26	409 ± 23
Metals and metalloids					
Chromium (Cr)	mg/L	1.50	3.34 ± 0.28	<L.Q. (0.75)	<L.Q. (0.75)
Other parameters for analysis and reporting					
Acidity	mg/L CaCO_3_	A&R	12.50 ± 1.7	8.23 ± 0.1	7.21 ± 0.1
Alkalinity	mg/L CaCO_3_	A&R	162 ± 15	4.42 ± 0.53	5.37 ± 0.7
Calcium hardness	mg/L CaCO_3_	A&R	65.04 ± 2.1	43.64 ± 1.3	43.75 ± 1.1
Hardness	mg/L CaCO_3_	A&R	51.34 ± 1.7	44.68 ± 1.5	44.56 ± 1.4
True color (absorbance measurements at different wavelengths: 436, 525, and 620 nm)	m^−1^	A&R	1.31 ± 0.06	0.025 ± 0.004	0.034 ± 0.003
			0.148 ± 0.009	0.002 ± 0.001	0.003 ± 0.001
			0.078 ± 0.003	0.001 ± 0.001	0.002 ± 0.001
Additional parameters of interest (not included in Colombian environmental regulations)					
Total organic carbon (TOC)	mg/L C	N.A.	39.15 ± 4.2	7.09 ± 0.27	7.11 ± 0.14
Conductivity	*µ*S/cm	N.A.	1720 ± 53	2015 ± 53	2093 ± 74
Hexavalent chromium (Cr(VI))	mg/L	N.A.	<L.Q. (0.1)	<L.Q. (0.1)	<L.Q. (0.1)
Oxygen dissolved	mg/L	N.A.	6.7 ± 0.2	6.8 ± 0.1	6.7 ± 0.1
Apparent color	U Pt-Co	N.A.	2721 ± 23	40 ± 14	49 ± 7
Color (dye concentration)	mg/L	N.A.	36 ± 0.5	0.8 ± 0.05	0.9 ± 0.07
Color	RGB scale	N.A.	[224; 211; 76]	[255, 255, 250]	[254; 254; 248]
	Tonality	N.A.			

L.Q., quantification limit; N.A., not applicable; A&R, analysis and report.

**Table 2:** Matrix of the experimental design and results of the response variables.

Test	Factors			Response variables (effluent of the Fenton process)		Response variables (effluent after neutralization pH = 6)	
	H_2_O_2_ mM	Fe^2+^ mM	pH initial	R_CN_	COpT	R_CN_	COpT
				(%)	(USD/m^3^)	(%)	(USD/m^3^)
1	16	1.15	5	95.74	0.0184	96.89	0.0184
2	2	2	5	97.48	0.0179	95.38	0.0179
3	16	2	7	36.06	0.0255	36.34	0.0255
4	30	2	5	98.52	0.0332	95.84	0.0332
5	30	1.15	3	98.12	0.0261	94.22	0.0261
6	2	1.15	3	97.03	0.0108	95.41	0.0108
7	30	1.15	7	21.04	0.0260	30.88	0.0261
8	2	0.3	5	21.80	0.0037	21.37	0.0037
9	16	0.3	3	97.57	0.0113	94.55	0.0113
10	16	2	3	96.93	0.0256	97.27	0.0256
11	2	1.15	7	14.77	0.0107	22.53	0.0108
12	16	0.3	7	3.73	0.0113	3.93	0.0113
13	16	1.15	5	98.21	0.0184	95.15	0.0184
14	16	1.15	5	97.98	0.0184	96.23	0.0184
15	30	0.3	5	18.93	0.0190	18.93	0.0190

**Table 3:** Analysis of variance for the %R_CN_ polynomial model (%).

Source	Sum of square	Degree of freedom	Mean square	*F* value	*p* value
A: pH	12328.4	1	12328.4	44.66	0.0002
B: [Fe^2+^]	4369.26	1	4369.26	15.83	0.0041
C: [H_2_O_2_]	3.82261	1	3.82261	0.01	0.9092
AA	1490.25	1	1490.25	5.40	0.0486
BB	1283.92	1	1283.92	4.65	0.0631
CC	1401.12	1	1401.12	5.08	0.0543
Error total	2208.25	8	276.031		
Total (corr.)	22529.2	14			
	*R* ^2^ = 91.45		*R* ^2^_adj_ = 84.73		

**Table 4:** Analysis of variance for the COpT polynomial model (USD/m^3^).

Source	Sum of square	Degree of Freedom	Mean square	*F* value	*p* value
A: pH	1.125*E* − 8	1	1.125*E* − 8	18.00	0.0028
B: [Fe^2+^]	0.000404701	1	0.000404701	647522.00	0.0001
C: [H_2_O_2_]	0.00046818	1	0.00046818	749088.00	0.0001
AA	5.76923*E* − 10	1	5.76923*E* − 10	0.92	0.3648
BB	5.19231*E* − 9	1	5.19231*E* − 9	8.31	0.0204
CC	5.76923*E* − 10	1	5.76923*E* − 10	0.92	0.3648
Error total	5.*E* − 9	8	6.25*E* − 10		
Total (corr.)	0.000872904	14			
	*R* ^2^ = 99.99		*R* ^2^_adj_ = 99.99		

**Table 5:** Optimal operating conditions predicted for the Fenton process and its experimental validation.

Fe^2+^ mM	H_2_O_2_ mM	pH initial	Response variable	Predicted	Experimental	Color (RGB)
0.981	5.38	3.15	R_CN_ (%)	99.65	97.86 ± 0.5	255; 255; 250
			COpT (USD/m^3^)	0.0112	0.0112 ± 0.0003	

**Table 6:** Comparison of operating conditions and removal efficiencies using Fenton and Fenton-related processes for the treatment of industrial or synthetic (simulated) wastewater from leather dyeing stage.

Method	Effluent characteristics	Operating conditions	Removal efficiency	Reference
Fenton (with nano-graphene oxide)	Industrial wastewater pH = 4.3 COD = 15125 mg O_2_/L BOD_5_ = 6300 mg O_2_/L BOD_5_/COD = 0.41 [Cr] = 280 mg/L	FeSO_4_ = 0.8 mg/L H_2_O_2_ = 30 mg/L CaO = 5 mg/L Cost = N.R. Kinetics study = N.R.	COD = 99% BOD_5_ = 98% BOD_5_/COD = 0.52 [Cr] = 99%	[14]

Fenton-neutralization	Industrial wastewater pH = 7.0 COD = 163.1 mg O_2_/L BOD_5_ = 13.47 mg O_2_/L BOD_5_/COD = 0.08 Dye concentration = 36 mg/L	pH = 3.15 FeSO_4_ = 0.981 mM H_2_O_2_ = 5.38 mM Cost = 0.0112 USD/m^3^ *k*_TOC_ = 1.17 × 10^−3^ (L.mg^−1^)^1.80^ min^−1^ *k*_CN_ = 1.52 × 10^−4^ (L.mg^−1^)^4.13^ min^−1^	COD = 82% TOC = 92% BOD_5_ = 98% BOD_5_/COD = 0.3 [Cr] > 99% (<L.Q. = 0.75 mg/L) Color = 97.5%	This study

Fenton heterogeneous	Industrial wastewater COD = 1920 mg O_2_/L BOD_5_ = 732 mg O_2_/L TOC = 650 mg C/L BOD_5_/COD = 0.38	Catalyst: activated carbon doped with Co oxide (1%) H_2_O_2_ = 10 mM pH = 3.5 *T* = 25°C Cost = N.R. *k*_COD_ = 6.22 × 10^−6^ L.mg^−1^min^−1^	COD = 77% = 441.6 BOD_5_ = 95% = 36.6 TOC = 80% BOD_5_/COD = 0.08	[35]

Photo-Fenton	Synthetic wastewater (Made of syntan and degreasing agents) COD = 100–350 mg O_2_/L Lethality = 100%	H_2_O_2_/FeSO_4_ = 150/500 (w/w) Toxicity tests = D. Magna *t* = 30 min pH = 3 *T* = 45°C rpm = 30 Cost = N.R. Kinetics study = N.R.	COD = 83% Toxicity = 90%	[36]

Electro-Fenton	Synthetic wastewater Mixture of tannery dyes (Violet RL-Green A) = 80 mg/L Na_2_SO_4_: 0.05 M (as support electrolyte)	Anode = BBD (2 cm^2^) Cathode = BBD (3 cm^2^) Electrode gap = 0.5 cm *t* = 60 min *j* = 50 mA/cm^2^ Fe^2+^ = 0.050 mM pH = 3 rpm = 500 Cost = N.R. *k*_color_ = 0.0949 min^−1^	Color = 99%	[71]
Photoelectro-Fenton	Synthetic wastewater Mixture of tannery dyes (Violet RL-Green A) = 80 mg/L Na_2_SO_4_: M (as support electrolyte)	Anode = BBD (2 cm^2^) Cathode = BBD (3 cm^2^) Electrode gap = 0.5 cm *j* = 50 mA/cm^2^ Fe^2+^ = 0.050 mM *t* = 60 min pH = 3 rpm = 500 Light source = UVA 365 nm Light intensity = 75 mA/cm^2^ Cost = N.R. *k*_color_ = 0.3480 min^−1^	Color = 100%	[71]

*j*, current density; *t*, operation time; *T*, temperature; *k*, rate constant; L.Q. = quantification limit; N.R., not reported.

## Data Availability

The data associated with this research are available from the corresponding author upon request.
